# Spin-Density Wave, Unconventional Magnetic and Thermal Transport Properties in Sr_2−*x*_(PbCl_2_)*_x_*Cu(BO_3_)_2_

**DOI:** 10.3390/ma17246179

**Published:** 2024-12-18

**Authors:** Dianta Ginting, Bora Won, Jong-Soo Rhyee

**Affiliations:** 1 Master of Mechanical Engineering Program, Faculty of Engineering, Universitas Mercubuana, West Jakarta 11650, Indonesia; 2 Department of Applied Physics, Institute of Natural Sciences, Kyung Hee University, Yongin 17104, Republic of Korea

**Keywords:** spin-density wave, Shastry-Sutherland lattice, spin gap, magnetization, heat capacity

## Abstract

SrCu_2_(BO_3_)_2_ (Sr-122) has attracted considerable interest as a quasi-two-dimensional S = 1/2 Heisenberg antiferromagnetic spin system with a Shastry-Sutherland lattice (SSL) structure. It features a Cu^2+^ spin dimer ground state and exhibits intra-dimer Dzyaloshinskii–Moriya interactions, making Sr-122 a fascinating platform for studying quantum magnetic phenomena. In this study, we investigate the β-phase of SrCu_2_(BO_3_)_2_ (β-Sr-212), which retains the same spin structure as Sr-122, to explore how the carrier concentration affects the spin gap. Our results show that increasing the doping levels in SrCu_2_(BO_3_)_2_ modulates the magnetic properties and slightly suppresses the spin gap, offering new possibilities for tuning its quantum magnetic behavior.

## 1. Introduction

The low-dimensional Heisenberg spin system is known for exhibiting exotic quantum states of matter [1]. This is particularly evident in strongly correlated oxides, such as La_2_CuO_4_ [2,3], Cu_2_Te_2_O_5_X_2_ (*X* = Br, Cl), and CaV_4_O_9_ [4], as well as in spin-ladder systems like Sr_2_CuO_3_ and SrCuO_2_ [5,6]. Among quasi-two-dimensional S = 1/2 Heisenberg antiferromagnetic spin systems, compounds featuring frustrated spin dimers, such as Ba_3_Mn_2_O_8_, Sr_2_Co_3_S_2_O_3_ [7,8,9,10], and SrCu_2_(BO_3_)_2_ (Sr-122), are of particular interest. These spin dimers often form a Shastry-Sutherland lattice (SSL), a two-dimensional square lattice with alternating diagonal bonds [11]. The Sr-122 compound in particular has a two-dimensional spin gap with a dimer ground state [12], and its magnetic interactions are heavily influenced by intra-dimer Dzyaloshinskii–Moriya interactions [13].

In the tetragonal structure of the Sr-122 compound (space group I-42m), the Cu^2+^ spins (S = 1/2) located at the edges of CuO_4_ units interact with other spins within the (BO_3_)_3_ octahedral units and those positioned in the ab-plane [14,15]. These Cu^2+^ (S = 1/2) spins form an SSL antiferromagnetic spin structure, characterized by spin doublet and triplet excitations [16,17]. Some researchers have proposed that the Sr-122 compound behaves as a Mott-Hubbard insulator [18].

The presence of a spin gap in Sr-122 has been detected through temperature-dependent magnetic susceptibility χ(T) and heat capacity C_p_(T) measurements, which are notably suppressed under an applied magnetic field [19]. Assuming that Sr-122 behaves as a Mott- or Mott-Hubbard-type insulator, previous studies have reported that doping can reduce the spin gap, potentially paving the way for superconductivity [20]. Theoretical investigations have proposed that superconductivity in an SSL system could manifest as a resonant valence bond (RVB) type with the introduction of carrier doping [21,22]. However, despite the suppression of the spin gap through doping with elements such as Al, La, Na, or Y at the Sr site, no superconductivity has been observed in the Sr-122 compound, contrary to theoretical predictions [19]. First-principles calculations suggest that the strong localization of the Cu d_x2−y2_ orbital near the Fermi level may account for the absence of superconductivity [23].

Sr_2_Cu(BO_3_)_2_ (Sr-212) is an analogous compound to SrCu_2_(BO_3_)_2_ (Sr-122); both feature an antiferromagnetic Shastry-Sutherland lattice (SSL) spin structure. The Sr-212 compound exhibits strong antiferromagnetic (AFM) ordering at a Néel temperature (T_N_) of 30 K [24]. The crystal and spin structures of Sr-212 differ significantly from those of Sr-122. In Sr-212, spin dimers are formed along the ac-plane by nearest-neighbor square Cu(2)O_4_ and octahedral CuO_6_ units, which are interconnected by triangular BO_3_ units [25,26].

Furthermore, Sr-212 features three distinct exchange interactions. Two octahedral Cu(1) units are connected by the exchange interaction J’ through B(2), while the Cu(1)-Cu(2) pairs are linked by J″ via B(1). The B(3)O_3_ unit lies between the Cu(1)-Cu(2) dimers, facilitating a dominant exchange interaction J, where J ≫ J′ or J″ [25].

In this study, we investigate the doping effect of PbCl_2_ in Sr_2−*x*_(PbCl_2_)*_x_*Cu(BO_3_)_2_ by substituting PbCl_2_ at the Sr site to increase the carrier concentration, with the aim of suppressing the spin gap. Our research focuses on the β-Sr-212 compound rather than Sr-122. This decision is motivated by the unique magnetic properties of β-Sr-212, which include a robust antiferromagnetic Shastry-Sutherland lattice (SSL) spin structure, pronounced spin-density wave behavior, and a well-defined spin gap. These attributes are crucial for examining how carrier doping influences magnetic interactions and suppresses the spin gap, which are central to our research objectives.

In contrast, Sr-212 does not exhibit the same level of tunability or the distinct magnetic behavior observed at low temperatures, making β-Sr-212 a more suitable candidate for exploring quantum magnetic phenomena. Our findings reveal the presence of spin-density wave behavior near 60 K, indicating the onset of long-range magnetic ordering at temperatures below 20 K. These results offer valuable insights into tuning the quantum properties in low-dimensional systems through strategic doping. Furthermore, they underscore the potential of β-Sr-212 as a platform for studying exotic magnetic behaviors and the interplay between spin-gap suppression and long-range order.

## 2. Materials and Methods

Polycrystalline samples of the β-phase Sr_2−*x*_(PbCl_2_)*_x_*Cu(BO_3_)_2_ (*x* = 0.0, 0.0005, 0.01, and 0.1) were synthesized using a solid-state reaction method [26]. Stoichiometric amounts of SrCO_3_ (99.994%, RND Korea, Gwangmyeong-si, Republic of Korea), PbCl_2_ (99.99%, RND Korea), CuO (99.995%, RND Korea), and B_2_O_3_ (99.98%, RND Korea) were thoroughly mixed and finely ground to ensure uniformity. Achieving consistent yields was critical, particularly for samples with small amounts of Cl doping, as variations could significantly affect the reproducibility and reliability of the magnetic properties. Across all doping levels, the average synthesis yield was 95% with a standard deviation of ±5%, demonstrating a controlled and reproducible synthesis process.

The finely ground powders were placed in a boat-shaped alumina crucible and heat-treated at 900 °C for 110 h, with periodic intermediate grinding to ensure homogeneity. Following the heat treatment, all samples exhibited a purple coloration, except for the sample doped with *x* = 0.1 PbCl_2_, which appeared dark purple. Each sample was subsequently hot-pressed at 700 °C (640 °C for the undoped sample) under a uniaxial pressure of 70 MPa for 1 h. The final densities of the samples exceeded 80% of their theoretical density. The final densities of the samples exceeded 80% of their theoretical density. Phase identification and lattice parameters were obtained by powder x-ray diffraction (XRD) measurements with Cu Ka radiation (D8 Advance, Bruker, Germany) performed on the hot-press sintered pellets for phase identification and determination of lattice parameters. The lattice parameters were calculated by using the Rietveld analysis.

The high-temperature sintering process resulted in polycrystalline particles with irregular morphologies, typical of solid-state reactions conducted at elevated temperatures. Such conditions generally promote particle agglomeration. The magnetic properties of the samples were measured using a Physical Property Measurement System (PPMS-Dynacool 14 T, Quantum Design, San Diego, CA, USA) with a vibrating sample magnetometer (VSM) option.

While the magnetic and structural properties of β-Sr_2−*x*_(PbCl_2_)*_x_*Cu(BO_3_)_2_ were the primary focus of this study, we acknowledge that particle size and morphology could also influence the observed phenomena. For instance, variations in particle size may impact magnetic interactions and the overall behavior of the material. To gain a more comprehensive understanding of the material’s properties, future research should include detailed analyses of particle size and morphology [27].

## 3. Results

**Figure 1:**
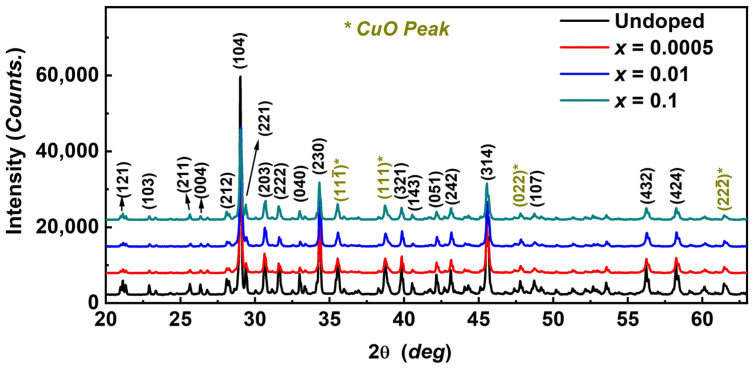
Powder X-ray diffraction patterns of Sr_2−x_(PbCl_2_)_x_Cu(BO_3_)_2_ (*x* = 0.0, 0.0005, 0.01, and 0.1) compounds.

Figure 1 presents the X-ray diffraction (XRD) patterns of Sr_2−*x*_(PbCl_2_)*_x_*Cu(BO_3_)_2_ at various doping levels (*x* = 0, 0.0005, 0.01, and 0.1). The undoped sample shows distinct peaks corresponding to the crystallographic planes of Sr_2_Cu(BO_3_)_2_. All observed peaks match the Sr-212 phase, along with a CuO impurity, which has been previously reported in studies on the Sr-122 compound [25]. The CuO impurities are commonly observed in Sr-122 compounds due to the non-stoichiometric loss of B_2_O_3_ (in gaseous form) during synthesis [26].

The appearance and growth of CuO peaks with increasing doping levels indicate the formation of secondary CuO phases [28,29]. Although XRD confirms the existence of CuO impurities, their impact on the magnetic properties is minimal. This is because the primary magnetic behaviors observed—such as spin-density wave and spin-gap characteristics—are intrinsic to the β-Sr-212 lattice. These phenomena are predominantly governed by the spin–spin coupling within the β-Sr-212 structure, and the antiferromagnetic nature of CuO does not significantly interfere with these interactions [28,29].

Moreover, our experimental findings are consistent with the magnetic properties previously reported for pure β-Sr-212, reinforcing the conclusion that CuO impurities have a negligible effect on the overall magnetic response [13,30].

Table 1 presents the calculated lattice parameters and lattice volume of polycrystalline Sr_2−*x*_(PbCl_2_)*_x_*Cu(BO_3_)_2_ for doping levels *x* = 0.0, 0.0005, 0.01, and 0.1. The lattice parameters of β-Sr-212 are a = 7.618 Å, b = 10.848 Å, and c = 13.522 Å, with an orthorhombic space group Pnma [29]. For small doping concentrations (*x* ≤ 0.01), the lattice parameters along the a-, b-, and c-axes exhibit a slight decrease as the doping level increases. Despite the larger ionic radius of Pb compared to Sr, the reduction in lattice volume implies an enhancement of bond strength due to PbCl_2_ doping.

Specifically, the undoped sample (*x* = 0) has lattice parameters of a = 7.618 Å, b = 10.848 Å, and c = 13.522 Å and a lattice volume of 1117.627 Å. When the doping concentration is increased to *x* = 0.0005, the a and b lattice parameters are slightly decreased, resulting in a reduced lattice volume of 1116.606 Å. At higher doping levels (*x* = 0.01 and *x* = 0.1), the lattice parameters show only minimal variation, remaining nearly constant within the measurement error margins. The lattice volume decreases to 1116.312 Å^3^ for *x* = 0.01 and then shows a marginal increase to 1116.882 Å at *x* = 0.1.

These observations suggest that PbCl_2_ doping has a relatively minor effect on the lattice parameters and volume, indicating that the crystal structure remains stable across the investigated doping range. Although the changes in lattice parameters are subtle, the increase in bond strength is significant enough to influence the magnetic interactions between Cu^2+^ ions. This enhanced spin–spin interaction can modulate the spin gap, demonstrating that even minor lattice distortions can impact the quantum properties of the material.

The hypothesis that PbCl_2_ doping enhances bond strength without significantly altering the lattice structure aligns with theoretical expectations observed in similar systems. Previous studies have demonstrated that ionic substitutions, such as the incorporation of Pb^2+^, can strengthen bonds through enhanced ionic interactions, even when the lattice parameters remain largely unchanged. This phenomenon is consistent with findings in Shastry-Sutherland lattice compounds, where structural rigidity is preserved despite doping [24].

**Figure 2:**
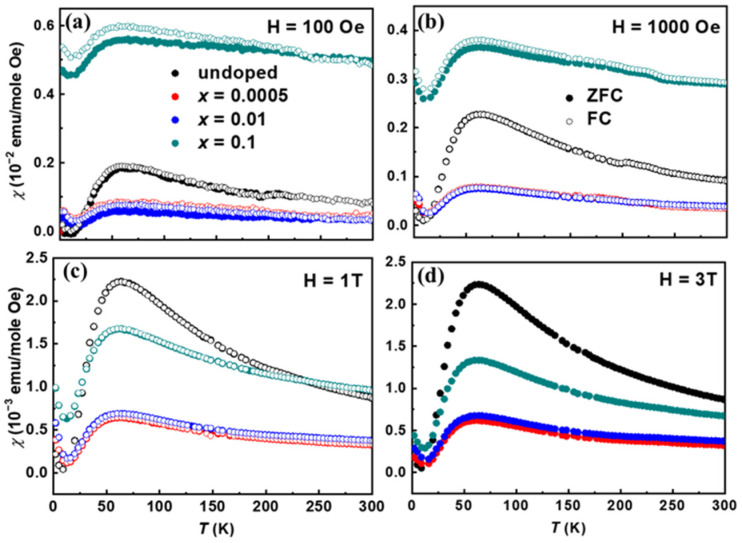
Zero-field-cooled (ZFC; closed circles) and field-cooled (FC; open circles) magnetic susceptibility as a function of temperature under magnetic fields of (**a**) 100 Oe, (**b**) 1000 Oe, and (**c**) 1 T. (**d**) Zero-field-cooled magnetic susceptibility for H = 3 T of Sr_2−x_(PbCl_2_)_x_Cu(BO_3_)_2_.

Figure 2 shows the magnetic susceptibility χ(T) of Sr_2−*x*_(PbCl_2_)*_x_*Cu(BO_3_)_2_ as a function of temperature from 2 K to 300 K, measured under zero-field-cooled (ZFC; closed circles) and field-cooled (FC; open circles) conditions at various magnetic fields. Thermal hysteresis between the ZFC and FC cycles was observed at low magnetic fields for all doping levels examined. The magnitude of this hysteresis varied with doping concentration, becoming more pronounced at higher doping levels, particularly for *x* = 0.1. This increase in hysteresis at elevated doping levels may be attributed to the enhanced ferromagnetic-like interactions introduced by PbCl_2_ doping, which could suggest a strengthened spin canting effect. The trends observed in the magnetic susceptibility measurements point to a potential spin canting phenomenon. This phenomenon has been theoretically described in systems subjected to perturbations that enhance magnetic interactions. For instance, intra-dimer Dzyaloshinskii–Moriya interactions and similar effects can induce spin canting, as noted in studies on Shastry-Sutherland lattice materials [31]. Our observations suggest that while hysteresis occurs across all doping levels, its impact becomes more pronounced at higher PbCl_2_ concentrations. The overall magnetic susceptibility value, χ, is approximately 10^−3^ emu mol^−1^ Oe^−1^, which is consistent with other reported results [23]. The data exhibit a broad maximum (T_max_) followed by a subtle minimum (T_min_) as the temperature decreases. The value of T_max_ slightly increases with increasing magnetic field. For example, in the undoped sample, T_max_ is observed at 48 K, 56 K, and 60 K for magnetic fields of H = 100 Oe, 1000 Oe, and 1 T, respectively, aligning with previous findings for single-crystalline β-Sr-212 [32]. The T_max_ value for single-crystalline β-Sr-212 is approximately 60 K, independent of the crystal orientation, consistent with our results [23]. The broad peak in magnetic susceptibility is attributed to a spin-gap state associated with the spin-density wave. Additionally, the χ(T) behavior of PbCl_2_-doped samples follows the spin-density wave pattern at temperatures T ≤ 60 K.

Figure 2a,b show the magnetic susceptibility χ(T) under low magnetic fields, highlighting the thermal hysteresis between field-cooled (FC) and zero-field-cooled (ZFC) cycles. As the magnetic field increases (H ≥ 1 T), the thermal hysteresis diminishes, and the χ(T) values for the ZFC and FC data converge, becoming nearly identical. The χ(T) values of lightly doped samples (*x* = 0.0005 and 0.01) are lower compared to the undoped sample (*x* = 0).

The spin canting phenomenon observed in Sr_2−*x*_(PbCl_2_)*_x_*Cu(BO_3_)_2_ with PbCl_2_ doping is driven by both structural and electronic factors. Substitution of Pb^2+^ ions at Sr^2+^ sites introduces localized lattice distortions, which enhance magnetic interactions among Cu^2+^ ions. These structural modifications strengthen spin–spin coupling, resulting in spin canting and the formation of ferromagnet-like dimers. At higher doping levels, especially for *x* = 0.1, these distortions become more pronounced, intensifying the canting effect.

Electronically, the introduction of PbCl_2_ alters the charge distribution within the lattice, further reinforcing the spin canting effect and contributing to the ferromagnetic-like behavior observed in heavily doped samples. This relationship between PbCl_2_ concentration and spin canting underscores the role of doping in tuning the quantum magnetic properties of Sr_2_Cu(BO_3_)_2_.

We also determined the key magnetic parameters, including the Curie constant (C) and Weiss temperature (*θ*). The effective magnetic moment (*μ_eff_*) per formula unit (*μ_B_*/f.u.) of polycrystalline Sr_2−*x*_(PbCl_2_)*_x_*Cu(BO_3_)_2_ (*x* = 0, 0.0005, 0.01, and 0.1) was calculated, as presented in Table 2. These parameters were obtained by fitting the Curie–Weiss law to the 1/χ vs. T data under a 1 T magnetic field across the temperature range of 150 K to 300 K.

Our results show that the Curie temperature increases proportionally with the PbCl_2_ doping concentration, except in the undoped case. For light doping levels (*x* ≤ 0.01), the Weiss constant θ significantly increases compared to the undoped sample, which exhibits antiferromagnetic (AFM) behavior. Despite these changes, T_max_ shifts only slightly with varying doping levels, likely due to the presence of a frustrated spin structure. The ratio between the Weiss temperature and the Néel temperature (θ/T_N_) reveals a significant spin frustration within the compound.

The θ/T_N_ ratio for doped samples is lower than that of the undoped sample, indicating that while spin frustration persists, it is somewhat suppressed by doping. Additionally, discrepancies were observed between the Weiss constant and T_min_ for each sample, likely due to spin fluctuations. For the undoped sample, T_min_ is 9.15 K at H = 1 T, with a Weiss temperature of 53 K. Similar discrepancies were noted for the PbCl_2_-doped samples, reinforcing the role of spin fluctuations in influencing magnetic behavior.

Table 3 summarizes the spin-gap values obtained from two independent measurements: zero-field-cooled magnetic susceptibility (Δ_1_) and zero-field specific heat (Δ_2_) for polycrystalline Sr_2−*x*_(PbCl_2_)*_x_*Cu(BO_3_)_2_ samples (*x* = 0, 0.0005, 0.01, and 0.1). The temperature-dependent magnetic susceptibility data were analyzed over a defined temperature range (T_min_ < T < T_max_). To improve the accuracy of spin-gap determination, the analysis incorporated additional terms beyond the standard exponential contribution.

The magnetic susceptibility versus temperature (χ-T) curves were analyzed using an established model previously applied to Sr-122 systems. This model incorporates the Curie–Weiss parameters—Curie constant (C) and Weiss temperature (θ)—as detailed in Table 2 for each doping concentration. The fitting equation consists of two main components: a Curie–Weiss term, which describes the paramagnetic behavior, and an exponential term, which characterizes the evolution of the spin gap (Δ) in Kelvin (K). Additionally, a term χ_0_ is included to account for the diamagnetic contribution from the ionic cores as follows:

(1) $$\chi =\frac{C}{T-\Theta }+aexp\left(-\frac{\Delta_{1}}{T}\right)+\chi_{0}$$

The calculated Δ_1_ values exhibit a non-linear trend with increasing doping concentration. Specifically, the spin gap initially increases from 32.2719 K to 33.7485 K and then decreases to 32.1197 K at the highest doping level. These values are comparable to those reported for β-Sr-122, a known antiferromagnetic material with a Néel temperature of 30 K and spin-gap values ranging from 30 K to 34 K [24,30]. For the undoped compound (*x* = 0), our measurements indicate a spin gap of Δ_1_ = 32.2719 K under a 1 T magnetic field, which progressively decreases to 31.8948 K under a 7 T field.

The spin gap shows non-monotonic behavior with PbCl_2_ doping, peaking at *x* = 0.01 and then declining at higher concentrations (*x* = 0.1). Notably, all samples achieve their maximum spin-gap values when subjected to a 3 T applied field.

The spin gap Δ_2_ can also be determined by fitting the specific heat data in the T < 7.5 K temperature range using the following equation [19]:

(2) $$C_{p}=\gamma T+\beta T^{3}+aexp\left(-\frac{\Delta_{2}}{T}\right)$$

The first and second terms of Equation (2) represent the electronic and phonon contribution of heat capacity at low temperatures, where *γ* and *β* correspond to the Sommerfeld coefficient and phonon contributions to the specific heat, respectively. We verified that the phonon contribution is minimal and negligible compared to the electronic term. For example, in the most heavily doped sample, *γ* is approximately 5.55 × 10^−3^ J/mol·K, and *β* is 4.27 × 10^−5^ J/mol·K^3^. The exponential term is dominant due to the coefficient *a*, which is 1.69 J/mol·K for the *x* = 0.1 sample.

The magnetization and heat capacity measurements indicate that the spin-density wave is suppressed with increasing doping concentration. As the magnetic field and doping levels increase, a slight decrease in the spin gap is observed. Conversely, the spin gap Δ_2_, derived from specific heat data without an applied magnetic field, increases by approximately 1 K with higher doping levels.

The spin-gap state in the PbCl_2_-doped β-Sr-212 compound is not significantly suppressed by the applied magnetic field or increased doping. However, a modest decrease of about 1 to 2 K in Δ is noted for all doped samples, except for *x* = 0. While this suppression is not drastic, it suggests the possibility of a phase transition from the spin state to a superconducting state.

**Figure 3:**
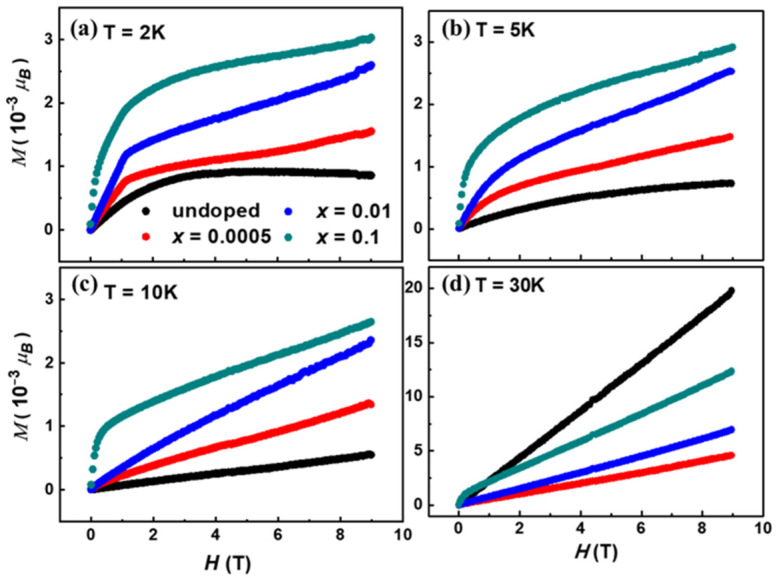
The magnetization as a function of field at (**a**) 2 K, (**b**) 5 K, (**c**) 10 K, and (**d**) 30 K with different samples of Sr_2−*x*_(PbCl_2_)*_x_*Cu(BO_3_)_2_.

Figure 3 shows the magnetization (M), expressed in units of 10^−3^ Bohr magnetons, for Sr_2−*x*_(PbCl_2_)*_x_*Cu(BO_3_)_2_ (*x* = 0, 0.0005, 0.01, 0.1) as a function of the applied magnetic field. The magnetization measurements were conducted at temperatures of 2 K, 5 K, 10 K, and 30 K using a Physical Property Measurement System (PPMS), which also controlled the applied magnetic field. The compound exhibits spin canting due to PbCl_2_ doping, leading to the formation of ferromagnetic (FM)-like dimers composed of pairs of Cu^2+^ ions. The suppression of these FM-like dimers is highly temperature-dependent, with the breakpoints shifting to lower fields as the temperature increases. Specifically, for *x* = 0.1, the breakpoints occur at approximately H = 1.1 T, 0.7 T, 0.6 T, and 0.45 T at 2 K, 5 K, 10 K, and 30 K, respectively.

For the undoped sample, magnetization increases with the applied magnetic field up to 3.8 T and then weakly decreases for H > 3.8 T at T = 2 K. This unconventional behavior has been previously observed in Sr-212 under high magnetic fields [23,33,34,35,36]. Such a phenomenon suggests the potential presence of superparamagnetic properties in the compound, akin to those seen in γ-Fe_2_O_3_ nanoparticles [33]. Additionally, β-Sr-122 exhibits a diamagnetic background [23], similar to Sr-122, which has a diamagnetic background term, *χ*_0_ [19].

In the doped samples, changes in slope are observed in the magnetization curves. For *x* = 0, 0.0005, and 0.01, the breakpoints occur at approximately H = 1.6 T. In contrast, for *x* = 0.1, the breakpoint is decreased at H = 7000 Oe, with additional slope changes appearing around H = 6000 Oe for the most heavily doped sample. At temperatures below 10 K, the *x* = 0.1 sample exhibits Brillouin-type magnetization behavior.

Notably, the magnetization curve for *x* = 0.1 sharply increases at low applied fields, a trend that persists over a wide temperature range. This behavior aligns with the observation that χ(T) for *x* = 0.1 is higher than that of the other samples (refer to Figure 2). In lightly doped samples (x ≤ 0.01), ferromagnetic (FM) properties disappear at T = 10 K, whereas FM-like behavior is still observed in the *x* = 0.1 sample.

At 30 K, the undoped sample shows the highest magnetization, followed by the *x* = 0.1 sample. The overall magnetization magnitude is preserved in the PbCl_2_-doped samples, consistent with the observed increase in χ(T) for *x* = 0.1. Notably, slope changes are only detected in the most heavily doped sample, with a breakpoint occurring around H = 4500 Oe. These findings suggest that ferromagnetic (FM) behavior is retained at relatively high temperatures (T = 50 K) exclusively when *x* = 0.1. As previously discussed, for doping levels x ≤ 0.01, FM properties disappear at T = 10 K.

**Figure 4:**
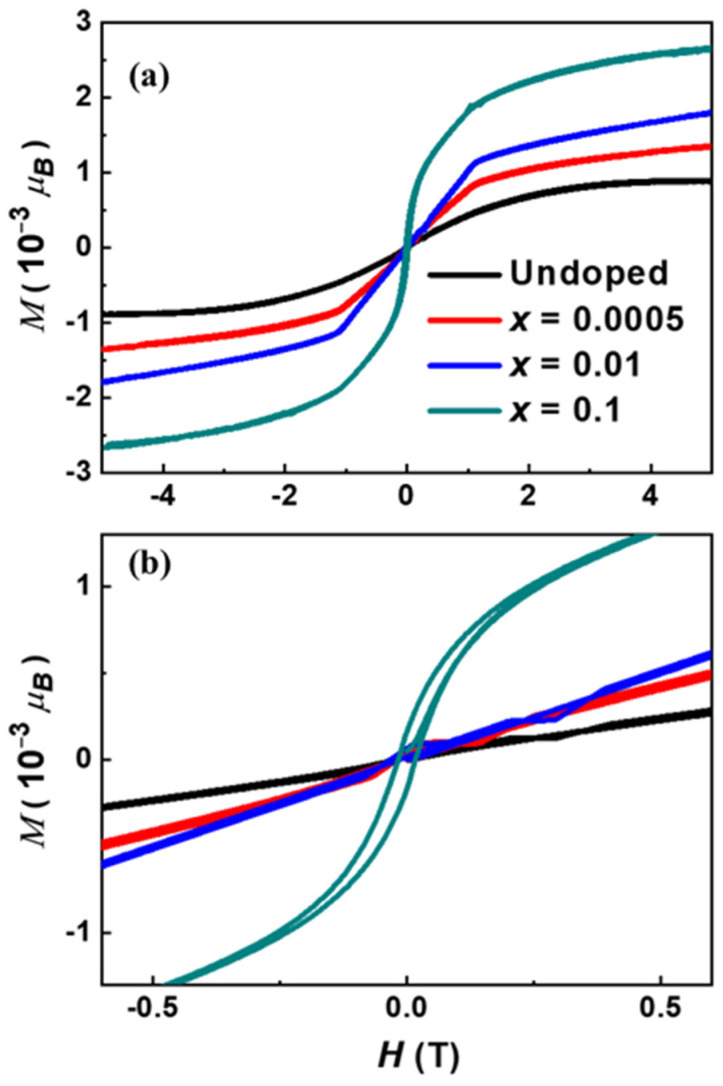
Magnetization as a function of applied field with different samples at T = 2 K in a field range of (**a**) −5 T < H < 5 T and (**b**) −0.6 T < H < 0.6 T.

Figure 4 illustrates the magnetization as a function of the applied magnetic field at a low temperature of T = 2 K. The magnetization is measured in units of 10^−3^ Bohr magnetons. The graphs exhibit step-like discontinuities within the |H| < 1 T range. Typical Brillouin-type behavior is observed in both the undoped sample and the most heavily doped case (*x* = 0.1), which exhibit paramagnetic properties. In contrast, the samples with intermediate doping levels (*x* = 0.0005 and 0.01) display characteristic antiferromagnetic (AFM) behavior.

For the *x* = 0.1 case, a coercive field was observed, along with a noticeable change in slope. This experimental finding suggests that the highest PbCl_2_-doped sample exhibits a hysteresis loop and a ferromagnetic (FM) phase, likely due to impurity effects. This observation is consistent with the magnetic susceptibility data, which revealed FM-like dimer behavior exclusively in the *x* = 0.1 samples. No hysteresis was observed in samples with lower doping levels or in the undoped sample, indicating reduced spin frustration compared to the *x* = 0.1 case.

This result is further corroborated by the hysteresis seen between field-cooled (FC) and zero-field-cooled (ZFC) curves in the χ(T) data. Additionally, the Weiss temperature for *x* = 0.1 is lower than that for *x* = 0.01, as shown in Table 2. The effective magnetic moment for *x* = 0.1 also increases compared to samples with lower doping levels. We propose that the presence of Cu^3+^ ions may contribute to weak FM coupling, as the most heavily doped sample exhibits mixed valence states of Cu^2+^ and Cu^3+^, unlike the other samples.

**Figure 5:**
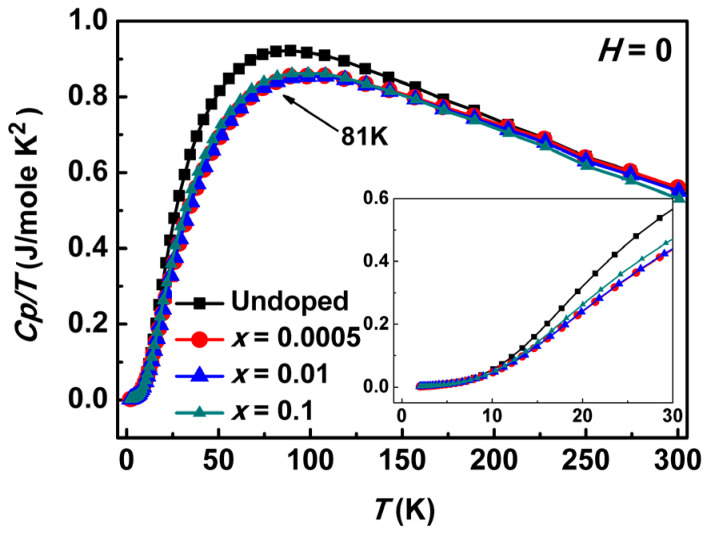
Heat capacity as a function of temperature range from 0 K to 300 K under zero magnetic field.

Figure 5 illustrates the specific heat of polycrystalline Sr_2−*x*_(PbCl_2_)*_x_*Cu(BO_3_)_2_ (*x* = 0, 0.0005, 0.01, and 0.1) as a function of temperature. Overall, PbCl_2_ doping suppresses the heat capacity. The inset displays the data within the temperature range of 2 K < T < 30 K, revealing a quadratic trend with positive curvature at low temperatures (T < 30 K). This behavior suggests that the heat capacity of Sr_2−*x*_(PbCl_2_)*_x_*Cu(BO_3_)_2_ samples is consistent with spin-density wave phenomena.

This observation aligns with the magnetic susceptibility data shown in Figure 3, which also indicates spin-density wave behavior within the T_min_ < T < T_max_ range. The heat capacity trends can be explained by a combination of electronic, phononic, and spin-density wave contributions, as described by Equation (2). Furthermore, the magnetic susceptibility results support the presence of a spin gap. The χ-T data follow the relation χ~exp (−Δ/k_B_T), but do not exhibit exponential decay as the temperature approaches zero. Consequently, the samples cannot be classified as spin-Peierls systems. Instead, χ increases slightly to a positive value below T_min_. This behavior cannot be attributed to magnetic impurities, such as CuO, since it has also been reported for crystalline β-Sr-212.

## 4. Conclusions

We conducted chemical doping experiments on polycrystalline β-phase Sr_2_Cu(BO_3_)_2_ by substituting Sr with PbCl_2_ to form Sr_2−*x*_(PbCl_2_)*_x_*Cu(BO_3_)_2_. The XRD data confirmed that the lattice parameters changed proportionally, indicating the successful incorporation of Pb atoms in place of Sr atoms. Using a vibrating sample magnetometer (VSM) on a physical property measurement system (PPMS), we observed unconventional magnetic behavior. The magnetic susceptibility of Sr_2−*x*_(PbCl_2_)*_x_*Cu(BO_3_)_2_ (*x* = 0, 0.0005, 0.01, and 0.1) exhibited spin-density wave behavior at temperatures T ≤ 60 K, with a pronounced minimum around T~13 K across all samples.

Interestingly, ferromagnetic-like properties were observed in the *x* = 0.1 sample, despite β-phase Sr_2_Cu(BO_3_)_2_ being typically antiferromagnetic. This behavior is likely due to the presence of a spin-1 Heisenberg model with mixed valence states of Cu^2+^ and Cu^3+^ in Sr_1.9_(PbCl_2_)_0.1_Cu(BO_3_)_2_. Although PbCl_2_ doping induced notable and unconventional magnetic properties, the compound remained electrically insulating, suggesting that hole doping did not significantly affect the localized electrons. The spin gap, determined from specific heat and magnetic susceptibility measurements, showed minimal suppression with increasing magnetic field and PbCl_2_ doping concentration.

Further studies, including additional chemical doping experiments, are necessary to explore the potential for superconductivity in these materials.

## Figures and Tables

**Table 1:** Lattice parameters and lattice volumes of Sr_2−x_(PbCl_2_)_x_Cu(BO_3_)_2_.

Doping (*x*)	*a* (Å)	*b* (Å)	*c* (Å)	*V* (Å^3^)
0.0	7.618(8)	10.848(5)	13.522(0)	1117.627(8)
0.0005	7.614(8)	10.845(4)	13.520(6)	1116.606(2)
0.01	7.614(3)	10.845(1)	13.518(3)	1116.312(1)
0.1	7.617(2)	10.848(2)	13.516(2)	1116.882(9)

**Table 2:** Curie constant, Weiss temperature, and effective momentum per formula unit of Sr_2−x_(PbCl_2_)_x_Cu(BO_3_)_2_.

Doping (*x*)	*C* (K·Emu/Mole-Oe)	*θ* (K)	*μ_eff_* (*μ_B_*)
0	0.31	−53.04	1.57
0.0005	0.17	−211.65	1.15
0.01	0.23	−306.17	1.34
0.1	0.54	−267.67	2.08

**Table 3:** Calculated spin gap deduced from the susceptibility ($\Delta_{1}$) and specific heat data ($\Delta_{2}$) by fitting Equations (1) and (2) of Sr_2−x_(PbCl_2_)_x_Cu(BO_3_)_2_.

Doping (*x*)	$\Delta_{1}$(*H* = 1 T) (K)	$\Delta_{1}$(*H* = 3 T) (K)	$\Delta_{1}$(*H* = 5 T) (K)	$\Delta_{1}$(*H* = 7 T) (K)	$\Delta_{2}$(*H* = 0) (K)
0	32.271(9)	32.937(5)	32.183(6)	31.894(8)	32.042(9)
0.0005	33.302(8)	34.337(7)	33.295(9)	32.221(9)	37.541(0)
0.01	33.748(5)	34.548(4)	33.720(8)	33.058(5)	37.529(7)
0.1	32.119(7)	32.863(8)	32.440(8)	32.296(9)	36.317(5)

## Data Availability

The original contributions presented in the study are included in the article, further inquiries can be directed to the corresponding authors.
